# Using the Family Planning Estimation Tool (FPET) to assess national-level family planning trends and future projections for contraceptive prevalence and associated demand for HIV-infected women in sub-Saharan Africa

**DOI:** 10.1371/journal.pgph.0002637

**Published:** 2024-08-06

**Authors:** Preshit Nemdas Ambade, Julia Hajjar, Nicholas Kofi Adjei, Sanni Yaya

**Affiliations:** 1 Department of Health Management, Economics and Policy, School of Public Health, Augusta University, Augusta, Georgia, United States of America; 2 Faculty of Health Sciences, Interdisciplinary School of Health Sciences, University of Ottawa, Ottawa, Canada; 3 Department of Public Health, Policy and Systems, University of Liverpool, Liverpool, United Kingdom; 4 The George Institute for Global Health, Imperial College London, London, United Kingdom; Western University of Health Sciences, CANADA

## Abstract

The combination of low uptake of modern contraceptives, high rates of unintended pregnancies, and the pervasive HIV epidemic in Sub-Saharan Africa (SSA) poses a threat to maternal, newborn, and child health in the region. This study examined the prevalence, need, and demand satisfied by modern contraceptive methods for women who tested positive for HIV (both unmarried and married) in 10 countries in SSA. We used the Family Planning Estimation Tool (FPET) to generate national-level trends and projections from 1983 through 2030. Individual-level data from 30 DHS surveys conducted between 2004 and 2018 in 10 sub-Saharan Africa (SSA) countries were used to produce projections for 1) all women and 2) unmarried and married women who tested positive for HIV. Throughout the period, Ethiopian and Guinean women who tested positive for HIV had a higher %mCPR (utilization of modern family planning methods) vis-à-vis all women. Among women who tested positive for HIV, the highest percentage of family planning demand satisfied by modern methods was observed in Zimbabwe (85.27, CI: 76.32−91.69), Lesotho (82.75, CI: 71.80−89.86), Rwanda (80.17, CI: 70.01−87.62), Malawi (73.11, CI: 61.50−82.63), and Zambia (72.63, CI: 64.49−80.09). The highest unmet need for modern contraceptives was found in Senegal (25.38, CI:18.36−33.72), followed by Cameroon (23.59, CI:19.30−28.59) and Sierra Leone (23.16, CI:16.64−32.05). Zimbabwe had the lowest unmet need (10.61, CI:6.36−16.13) and achieved the highest change in %mCPR (49.28, SE:6.80). Among married women who tested positive for HIV, their unmet need for modern contraception will remain higher in 2030. Continuing existing policies until 2030 would result in significant coverage gain among married vis-à-vis unmarried women who tested positive for HIV. Our projections emphasize the importance of country-specific strengthening initiatives, programs, and services targeting unmarried women.

## Background

### Family planning and HIV in SSA

Despite improvements in modern contraceptive uptake in the past decade, there remains an immense subset of the population in low- and middle-income countries (LMICs) that do not receive equitable access to sexual and reproductive health services [1]. Over a billion women and girls of reproductive age (15–49) reside in LMICs, and among them, 371 million use modern forms of contraception [2]. The rate of unintended pregnancies among women of reproductive age is notably higher (91 per 1000) in sub-Saharan Africa (SSA) than in the Western world (35 per 1000) [3]. Unintended pregnancies can result in a myriad of severe and often life-threatening health consequences, including unsafe abortion and maternal mortality [4]. According to the United Nations (UN), modern contraceptive uptake can be defined as the use of any form of modern contraceptive method [5]. The latest UN 2020 World Fertility and Family Planning report revealed that the highest rates of contraceptive uptake were observed in East Asia and South Asia (60%), followed by Latin America and the Caribbean, North America and Europe, and Australia and New Zealand, with 58% of women using contraceptives in these regions, respectively [5]. Notably, Sub-Saharan Africa (SSA) had the second lowest rate of contraceptive uptake in 2019, at 29%, after Oceania at 28% [5], and has the lowest contraceptive uptake among developing regions, mainly due to limited access to family planning resources [6]. Ensuring the availability of accessible and appropriate family planning services is therefore vital in mitigating the often-deadly consequences of unintended pregnancies, particularly for women in SSA [4]. As of 2021, about 66% of the world’s HIV-positive population resides in the WHO African region [7]. In SSA, there are 25.7 million individuals living with HIV. However, only 81% are aware of their HIV status, and 64% can access anti-retroviral therapy (ART) [8]. This highlights a significant gap in HIV education and service accessibility. In addition to the low uptake of family planning methods in SSA, HIV infections disproportionally affect women and girls in the region, posing a significant health equity concern [9,10]. In 2020, females aged 15–24 accounted for one-quarter of all HIV infections in SSA [10], placing them at heightened risk for adverse lifelong sexual and reproductive health consequences. Thus, the HIV epidemic in SSA, combined with low modern contraceptive uptake and high rates of unintended pregnancies, presents a significant health equity issue and poses a dire threat to maternal, newborn, and child health. Patriarchal culture and gender inequities can result in low health autonomy and health decision-making for women and girls [11,12]. This renders them particularly vulnerable to risks associated with unintended pregnancies and HIV [13]. To enhance the health and quality of life for people living in SSA, especially young women, prioritizing the integration of family planning and HIV services is of paramount importance. The Family Planning Estimation Tool (FPET) generates annual projections of family planning needs in countries around the world [14]. It can provide valuable foresight for countries to anticipate gaps in services better and identify specific subpopulations that would benefit from targeted programs and services. This tool would be invaluable in the fight against HIV/AIDS in SSA.

### The Family Planning Estimation Tool (FPET)

The FPET is used by countries to inform their national and sub-national strategies for family planning and to monitor the FP2020 initiative by generating projections of family planning needs [14]. The tool produces yearly projections of modern methods of contraceptive prevalence rate (mCPR), contraceptive prevalence rate (CPR), i.e., the percentage of women currently using any family planning method, in addition to projecting the unmet need for family planning and demand satisfied by modern methods [15]. Previous studies have utilized the FPET to generate projections for family planning on a global scale. For instance, the tool has been used to generate projections for contraceptive prevalence and unmet need for family planning in 194 countries [15], to estimate levels and trends of modern contraceptive prevalence rates, unmet need, and demand satisfaction in 436 sub-national areas across 26 countries in SSA [16], as well as 29 states and union territories in India [17]. The FPET tool utilizes a revised definition of unmet need for family planning [18] that excludes the calendar data from Demographic and Health Surveys (DHS) for calculation [19]. Previously, the unmet need calculation included calendar data whenever available and a different algorithm to calculate the need when such data was not provided. This approach led to unreliable estimates across the surveys, even within a country. The new definition provides more reliable and consistent estimates that are comparable across surveys and countries [19]. Our study applied the FPET to estimate national trends and create projected data starting from 1983, the onset of the HIV-AIDS epidemic in Africa, up until the year 2030. Specifically, we used the FPET to examine the prevalence, need, and demand satisfied by modern contraceptive methods among unmarried and married women who resided in 10 SSA countries and tested positive for HIV. These projections allowed us to identify target groups needing additional support, anticipate service gaps, and identify specific needs for countries and subgroups (i.e., married vs unmarried women). These projections could inform the development of tailored mitigation strategies that could accurately address country-specific needs and proactively address future challenges.

### Global targets and initiatives

As part of the Joint United Nations Programme on HIV/AIDS (UNAIDS) “2025 UNAIDS 95-95-95” testing and treatment targets, HIV Services Target 1 aims to ensure that “95% of women of reproductive age have their HIV and sexual and reproductive health service needs met; 95% of pregnant and breastfeeding women living with HIV have suppressed viral loads; and 95% of HIV-exposed children are tested by 2025”[20]. The 2025 UNAIDS targets also emphasize integration, which involves providing comprehensive care for various demographic groups affected by HIV, including pregnant women, migrants and refugees, youth, and sex workers. This integrated care encompasses HIV services, intimate partner violence (IPV) and mental health resources, STI testing, and sexual and reproductive healthcare [20]. Education on healthy lifestyle choices is also prioritized as part of integrated care for people living with HIV [20].

Integrating family planning and HIV services is a crucial preventative approach that can proactively address gaps in care. This approach can help to support the achievement of Sustainable Development Goal 3 (SDG 3): “to ensure healthy lives and promote well-being for all at all ages” [21]. By mitigating unintended pregnancies, providing educational tools, and testing for family planning and HIV, this integration can ultimately reduce the vertical transmission of HIV from mothers to their children. Consequently, this would help reduce maternal, neonatal, and child mortality rates and would contribute to the global efforts of the World Health Organization (WHO) to combat the AIDS epidemic and other infectious diseases targeted for eradication by 2030 [21]. Notably, SDG 3 includes component 3.7, which aims explicitly to integrate reproductive health into national-level programs and provide universal access to sexual and reproductive health services, including education and family planning resources [21]. Service integration also aligns with SDG 5, which seeks to achieve gender equality and empower all women and girls [22], thus promoting improvements in this aspect as well.

**Fig 1:**
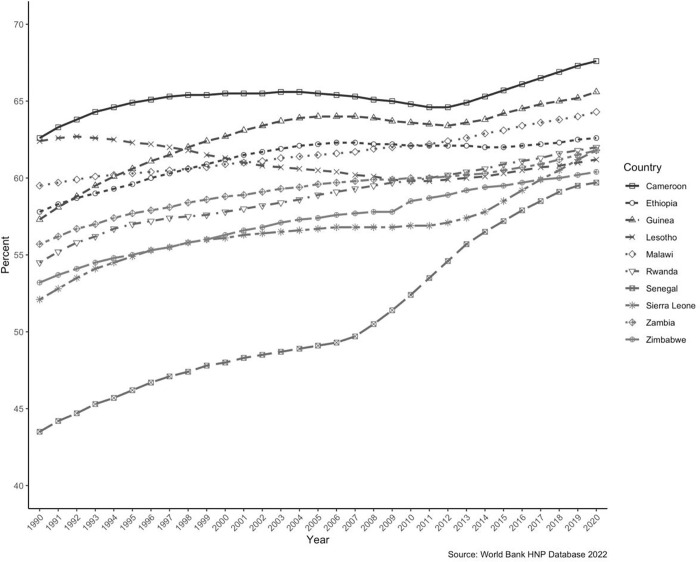
Women’s share of the population (both married and unmarried) ages 15+ living with HIV (%) for the selected Sub-Saharan African Countries.

The share of women who tested positive for HIV aged 15 years and above is ever-increasing in the selected SSA countries [23] (Fig 1). With the steady increase in this population subset, it is crucial to understand the current trend in family planning (FP) services utilized, the demand, and future needs. This paper aims to provide related insights and suggest future policy action items.

## Materials and methods

### Ethics statement

A formal request for analysis of all data was made to measure DHS through the online platform, and permission was granted (see S1 Text). The original data was collected with ethical approval from the ICF’s International Review Board.

### Data

Our analysis utilized individual-level data from 30 DHS surveys conducted between 2004 and 2018 in 10 SSA countries (see S1 Table). Further information regarding the DHS surveys can be accessed on the DHS website [24]. The selection of countries for our analysis was based on the criteria of having at least three data points to ensure reliable projections [14]. We included 30 DHS surveys, three from each country, as S1 Table shows. For each survey, we estimated the proportion of (un)married women who tested positive for HIV aged 15–49 years who were using at least one family planning method (modern and traditional). To assess the requirement for family planning services, we employed a revised definition of unmet need, which accounted for the proportion of women who did not desire any additional children or wanted to space out their next birth by at least two years but currently were not using any contraceptive method [19]. To ascertain the HIV-positive status of the women, we adhered to the definition provided by the DHS [25].

For each country, we determined the yearly population of HIV-infected women (15–49 years) using the AIM module of Spectrum software v6.1 [26]. For this purpose, we first imputed the 1970 age-group-wise population data for (un)married women into the DemProj module of the software. These base populations were derived using World Population Prospects 2019 [27] and Estimates and Projections of Women of Reproductive Age Who Are Married or in a Union: 2020 Revision [28]. No additional methodological changes were made to the software.

### FPET projections

The data analysis and projection estimation were done between January 2022 and December 2022. The country-level family planning data and yearly HIV population estimates were inputted into the FPET [14]. This online tool uses Bayesian Hierarchical modeling to provide annual estimates of mCPR, CPR, unmet need, and demand satisfied by modern methods. The tool utilizes logistic growth curves to fit contraceptive prevalence rates and determines time trends in contraceptive usage and related demand, as proposed in a previous study [15]. It allows data to be pulled across several surveys by assigning greater weight to the survey with lesser error variance. The recent changes in the tool allowed us to accommodate short-term changes in contraceptive prevalence, thus capturing the recent progress in family planning. The developer’s website provides more details on the tool and methodology [14].

Using the FPET, we estimated the national-level trend and future projections from 1983 up to 2030 for contraceptive prevalence and the associated demand for women who tested positive for HIV from the included countries in SSA. Mainly, the median values and associated 95% upper and lower bounds are presented. Our study placed particular emphasis on the prevalence, need, and demand satisfied by modern contraceptive methods following a previous study [17]. We showed separate results for unmarried and married women who tested positive for HIV as well as an overall estimate combining both groups. However, we mainly discussed the projections for all women samples, while the estimates for married (or in a union) and currently not married (or not in a union) data are presented in S2 Table. We also estimated changes in mPCR between 1983–2022 and 2022–2030, respectively. For the selected indicators, we followed the standard definitions provided in the UN’s World Contraceptive Use 2019 document [29].

## Results

**Fig 2:**
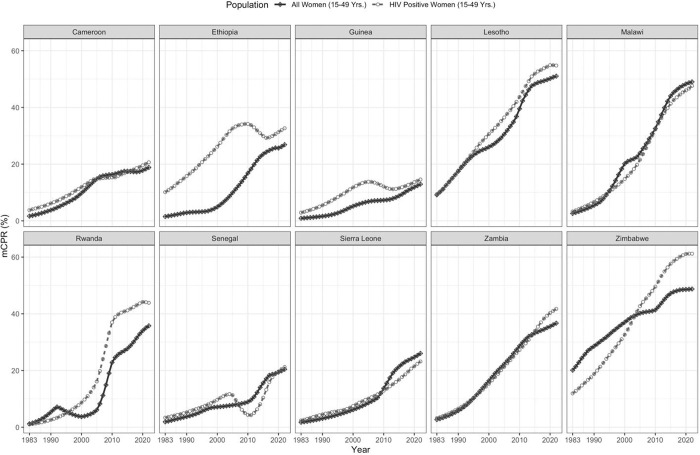
Trend in modern contraceptive prevalence (all-women versus women who tested positive for HIV) (% mPCR) for selected Sub-Saharan African Countries 1983–2022.

Fig 2 illustrates the national-level trends in modern contraceptive prevalence among women (15–49 yrs.), comparing the trends for all women to those of women who tested positive for HIV within the same age group from 1983 to 2022.

This figure shows a diverse pattern of growth in contraceptive prevalence across the countries under study. Notably, in Ethiopia, a substantial disparity in %mCPR was evident between women who tested positive for HIV and all women, with the former consistently exhibiting higher rates than the latter. Similarly, in Guinea, the utilization of modern family planning methods remained higher among women who tested positive for HIV compared to all women. In Lesotho, Rwanda, Zambia, and Zimbabwe, women who tested positive for HIV had surpassed all women in terms of their use of modern contraception over the past two decades. For the remaining countries, the difference in modern contraception prevalence rates between the two groups remained relatively similar and indistinguishable.

Table 1 presented the 2022 estimates and 2030 projections for mCPR, demand satisfied, and unmet need for modern contraceptives among women who tested positive for HIV. Significant variations regarding mCPR and other indicators could be seen across the selected countries. In 2022, Zimbabwe (61.21, CI:50.64−70.66) and Lesotho (54.78, CI:43.19−66.15) had the highest prevalence of modern family planning methods among women who tested positive for HIV. Conversely, Guinea (14.57, CI: 9.96−20.98) and Cameroon (20.64, CI:15.33−26.72) had the lowest prevalence of modern family planning methods. The percentage of family planning demand satisfied by modern methods among women who tested positive for HIV was highest in Zimbabwe (85.27, CI: 76.32−91.69), followed by Lesotho (82.75, CI: 71.80−89.86), Rwanda (80.17, CI: 70.01−87.62), Malawi (73.11, CI: 61.50−82.63), and Zambia (72.63, CI: 64.49−80.09). For the remaining countries, the demand satisfied by modern methods remained between 45 to 60 percent. In 2022, Senegal (25.38, CI:18.36−33.72) had the highest unmet need for modern contraceptives among women who tested positive for HIV, followed by Cameroon (23.59, CI:19.30−28.59) and Sierra Leone (23.16, CI:16.64−32.05). The lowest unmet need was observed for Zimbabwe (10.61, CI:6.36−16.13). The changes in mCPR between 1983 and 2022 across the SSA countries indicated an uneven growth in modern contraceptive prevalence among the study group. Zimbabwe (49.28, SE:6.80) led the pack to achieve the highest %mCPR change. Lesotho, Malawi, and Rwanda also recorded changes above 40 percent. The remaining countries experienced changes ranging from 11 to 39 percent.

**Table 1:** Prevalence and future projections of use of modern contraceptives along with their demand satisfied, unmet need for women who tested positive for HIV (15–49 yrs.) (both married and unmarried) residing in selected Sub-Saharan African Countries.

Country/ Population	Prevalence of Modern Methods (mCPR) (%), 2022	Demand Satisfied with a Modern Method (%), 2022	Unmet need for modern methods (%), 2022	Change in mCPR 1983-2022(SE)	Prevalence of Modern Methods (mCPR) (%), 2030	Demand Satisfied with a Modern Method (%), 2030	Unmet need for modern methods (%), 2030	Change in mCPR 2022-2030(SE)
Cameroon	20.64 (15.33−26.72)	46.56 (38.32−55.79)	23.59 (19.30−28.59)	16.89 (4.55)	25.16 (15.30−37.35)	52.70 (39.23−67.53)	22.17 (15.95−28.63)	4.52 (5.78)
Ethiopia	32.66 (23.80−42.05)	58.67 (46.38−70.87)	22.68 (16.70−30.05)	22.54 (6.44)	38.22 (23.81−52.35)	66.05 (47.22−81.07)	19.51 (11.84−28.92)	5.56 (6.84)
Guinea	14.57 (9.96−20.98)	49.02 (36.38−60.84)	15.33 (10.80−21.23)	11.68 (4.27)	18.84 (10.25−32.73)	53.00 (35.06−70.76)	16.37 (11.25−23.58)	4.27 (5.79)
Lesotho	54.78 (43.19−66.15)	82.75 (71.80−89.86)	11.48 (7.16−17.98)	45.38 (6.92)	51.54 (34.86−70.71)	84.85 (69.46−93.78)	9.03 (4.43−18.49)	-3.24 (7.67)
Malawi	47.62 (36.32−58.58)	73.11 (61.50−82.63)	17.53 (11.73−24.16)	44.33 (5.56)	52.30 (37.78−66.86)	78.15 (61.69−89.31)	14.66 (7.68−23.57)	4.68 (7.16)
Rwanda	43.89 (34.44−53.19)	80.17 (70.01−87.62)	10.87 (7.05−15.59)	42.91 (4.72)	45.19 (32.85−57.24)	81.90 (68.76−90.55)	10.06 (5.35−16.52)	1.31 (6.57)
Senegal	21.22 (14.28−29.98)	45.52 (33.09−58.82)	25.38 (18.36−33.72)	17.84 (5.06)	26.31 (15.55−40.90)	52.90 (36.59−70.60)	23.08 (15.19−32.52)	5.09 (6.41)
Sierra Leone	23.18 (15.87−32.06)	50.10 (35.48−63.24)	23.16 (16.64−32.05)	20.79 (4.74)	30.11 (17.34−46.52)	58.07 (39.87−75.66)	21.63 (14.04−31.62)	6.93 (6.74)
Zambia	41.74 (34.11−49.75)	72.63 (64.49−80.09)	15.56 (11.77−19.84)	38.54 (5.21)	46.79 (34.14−59.44)	76.72 (63.70−86.67)	14.21 (8.94−20.82)	5.05 (6.40)
Zimbabwe	61.21 (50.64−70.66)	85.27 (76.32−91.69)	10.61 (6.36−16.13)	49.28 (6.80)	61.90 (47.29−74.12)	86.64 (73.53−93.99)	9.56 (4.64−17.32)	0.70 (6.84)

Based on projections, it was anticipated that Zimbabwe and Lesotho would not experience a substantial increase in %mCPR compared to their rates in 2022. However, Sierra Leone (6.93, SE:6.74), Ethiopia (5.56, SE:6.84), Senegal (5.09, SE: 6.41), and Zambia (5.05, SE: 6.40) were expected to witness the highest increase by 2030. In the same years, Cameroon, Guinea, and Malawi could observe a prevalence increase of 4 percent compared to their rates in 2022. Rwanda’s growth would be approximately 1.31 times its estimate from 2022. By 2030, Cameroon, Ethiopia, Malawi, Senegal, Sierra Leone, and Zambia will experience a notable rise in the demand for modern methods of contraception (ranging between 4.52% and 6.93%) compared to 2022. If current strategies continue, no country will witness a significant reduction in the unmet need for modern methods by 2030. The unmet need would remain above 20% in Senegal, Cameroon, and Sierra Leone.

The disaggregated results for married and unmarried women who tested positive for HIV are shown in S2 Table. For the married group, Zimbabwe had the highest percentage of modern contraceptive prevalence rate (%mCPR) at 68.29% (CI: 55.31−79.69), followed by Lesotho, Rwanda, Malawi, and Zambia. For the rest of the countries, the prevalence of modern contraception was between 13% and 38%. The countries with the lowest prevalence rates experienced the highest unmet need for modern contraception among the married group. The changes in %mCPR between 1983 and 2022 across the countries were consistent with the overall estimates, suggesting that the rates among the married group significantly influenced the main findings. According to panel B of S2 Table, Lesotho (47.62, CI: 30.79−65.92) had the highest %mCPR for unmarried women, followed by Zimbabwe and Malawi. Like the results for married women, the countries with the lowest prevalence rates had higher unmet needs for modern contraception among unmarried women who tested HIV-positive.

The higher prevalence observed in 2022 aligned with the changes in the percentage of modern contraceptive prevalence rate (%mCPR) between 1983 and 2022 across all countries. It was anticipated that by 2030, all countries would experience a substantial increase in %mCPR for married women compared to the data from 2022. However, the prevalence among unmarried women who tested positive for HIV would be expected to remain relatively stagnant, with only a slight 1–2 percentage point increase in a few countries. In comparison to the unmarried population, the unmet need for modern contraception among married women who tested positive for HIV was likely to remain higher in 2030.

Among all countries, especially Cameroon (with an estimated unmet need of 27.86, CI: 19.55−37.34), Senegal (with an estimated unmet need of 27.85, CI: 18.00−39.06) would face the highest unmet need for modern contraception among the married group. Whereas Sierra Leone (with an estimated unmet need of 13.35, CI: 5.45−27.32) would face the highest demand for the unmarried groups. If countries continue their existing policies and programs until 2030, it is more probable that they will achieve significant coverage among HIV-positive married women compared to their unmarried counterparts.

## Discussion

Our findings indicated that women who tested positive for HIV in Ethiopia and Guinea had higher rates of utilization of modern contraception compared to all women. In Lesotho, Rwanda, Zambia, and Zimbabwe, women who tested positive for HIV surpassed all women in their use of modern contraception over the past two decades. Zimbabwe and Lesotho had the highest prevalence of modern family planning methods among women who tested positive for HIV, while Guinea and Cameroon had the lowest prevalence of modern family planning methods. The highest percentage of family planning demand satisfied by modern methods among women who tested positive for HIV was found in Zimbabwe, followed by Lesotho, Rwanda, Malawi and Zambia. In 2022, Senegal had the highest unmet need for modern contraceptives among women who tested positive for HIV, followed by Cameroon and Sierra Leone. Zimbabwe achieved the highest %mCPR change from 1983–2022, followed by Lesotho, Malawi and Rwanda. Sierra Leone, Ethiopia, Senegal, and Zambia were expected to experience the highest increase in %mCPR by 2030. By 2030, Cameroon, Ethiopia, Malawi, Senegal, Sierra Leone, and Zambia will experience a notable rise in the demand for modern contraception. In the results for both married and unmarried women who tested positive for HIV, the countries with the lowest prevalence rates for modern methods had a higher unmet need for modern contraception.

In women who tested positive for HIV, the utilization of family planning plays a vital role in reducing the occurrence of unplanned pregnancy and its subsequent consequences, such as the vertical transmission of HIV [30,31] as well as associated maternal and child mortality [32,33]. In SSA, several key factors contribute to unintended pregnancies among women who test positive for HIV, including inadequate knowledge and education regarding family planning and HIV transmission prevention [34]. To effectively address both family planning and HIV testing, treatment and care, the integration of services has emerged as a promising strategy to provide a comprehensive range of services alongside primary health services [35]. Integration aims to combine traditionally separate components of each service and optimize limited resources [35]. This strategy facilitates the ease of seeking care and improves accessibility to a wide array of sexual and reproductive health concerns in a single visit. Such integration is particularly vital in resource-limited regions with fragile healthcare infrastructure. The goal of integrating family planning and HIV services in SSA is to expand the range of easily accessible services available to patients at family planning clinics, encompassing STI testing and HIV-related services and support [32].

### Education

It has been shown that women are frequently compelled to forgo their educational pursuits due to early marriage [36]. This unfortunate circumstance often leads to untimely and unintended pregnancies, amplifying the risk of contracting HIV [37]. Furthermore, it increases the likelihood of resorting to unsafe abortions and experiencing maternal mortality [38]. Moreover, prevailing gender and sociocultural norms in Sub-Saharan Africa (SSA) contribute to men assuming the primary role in decision-making [11]. Consequently, this places women in inequitable positions within households and limits their autonomy over personal health matters. This phenomenon has been observed in rural Ethiopia [12] as well as in Senegal [39]. Therefore, advancing the consistent integration of services across SSA is crucial. This imperative action will ensure that integrated programs possess the capacity for scalability and ultimately contribute to achieving the SDGs 3 and 5 targets in the SSA region.

In this study, the prevalence of mCPR among women who tested positive for HIV was higher than all women in all countries except for Malawi and Sierra Leone; Fig 2. We hypothesized that the lower rate observed in Malawi may be attributed mainly to lower commodity supply and service availability issues, as revealed in a national assessment of implementation strength performed by Malawi’s National Evaluation Platform [40]. In Sierra Leone, the uptake of family planning services has remained subpar, and the country has the highest rate of maternal mortality and infant and child under five mortality rates globally [41]. In Fig 2, the mCPR % in Sierra Leone was slightly higher in HIV-positive women compared to all women from 1983–2010. However, in 2010, the mCPR % in all women surpassed that of women who tested positive for HIV. In the literature, factors identified as barriers to contraception use for young individuals (aged 10–24) in Sierra Leone included distance to a health facility, low literacy levels, the ability to ask their partner to use a condom, and boys’ knowledge of available contraceptive methods [42].

Conversely, Malawi has one of the highest total fertility rates in SSA [43] and one of the highest prevalence rates of HIV in SSA [44]. The Malawi Population HIV Impact Assessment 2020–2021 (MPHIA) report indicated that approximately 9% of individuals over the age of 15 in the country were living with HIV [45]. Furthermore, in Malawi, HIV prevalence rates are 10.8% and 6.4% for women and men, respectively [46]. Despite high HIV rates in the country, Malawi has been delivering effective HIV treatment initiatives and has already achieved targets 2 and 3 of the UNAIDS 95-95-95 targets [45]. Malawi has approximately 50% unmet contraceptive demand, resulting in high rates (41%) of unintended pregnancies [46]. Thus, we hypothesized that the lower mCPR prevalence among women who tested positive for HIV observed in Malawi in Fig 2 may be attributed to the high rates of unmet demand for contraception in the country. Another barrier identified by women who tested positive for HIV in Malawi is the lack of use (or inconsistent use) of male condoms during intercourse, mainly due to male decisions [47]. The high rates of HIV paired with lower mCPR prevalence in Malawi emphasizes the urgency of promoting service integration, including educational resources around family planning and HIV in high-need countries. It is evident that social determinants of health and other systemic factors continue to act as persistent barriers to delivering adequate sexual and reproductive health services in Malawi and Sierra Leone. This further emphasizes the urgent need for integration of family planning and HIV services in high-risk countries such as these. Driving forward integration initiatives would strengthen the spectrum of available services to mitigate unintended pregnancies and reduce transmission of HIV between partners and between mothers and their children to improve health and well-being for individuals residing in SSA.

### Examples of effective HIV prevention plans

In our study, Zimbabwe had the highest prevalence of modern family planning methods among women who tested positive for HIV, the highest family planning demand satisfied by modern methods, and the least unmet need. In 2021, Zimbabwe implemented the Zimbabwe National HIV and AIDS strategic plan 2021–2025 [48]. This initiative focused on both HIV and STI prevention and treatment and the integration of these services while addressing sociocultural barriers through strategies such as increasing male engagement [48]. This program encompassed a myriad of services, including the provision of condoms, pre-and post-exposure prophylaxis treatments, elimination of vertical transmission for HIV and STIs, and an HIV prevention program for teen girls and young women [48]. Notably, one of the primary foci of the 2021–2025 targets is to integrate family planning and HIV services for individuals in the country, particularly for women who tested positive for HIV during pregnancy [48]. This program highlights the importance of family planning and HIV service integration to address pervasive gaps that traditionally exist in siloed healthcare service formats.

### Exploring the unmet need for married women who tested positive for HIV

Notably, projections generated in our study using the FPET revealed that the higher unmet need for married women who tested positive for HIV will remain elevated in 2030. This finding was consistent with the higher unmet need for modern contraception in married women who tested positive for HIV identified in the literature. For example, a recent systematic review and meta-analysis in Ethiopia reported a prevalence of 25% for unmet need for family planning for women who tested positive for HIV [49]. Factors associated with unmet need included younger age (15–24 years old), formal non-literacy, lack of access to information about family planning, and not communicating about family planning with their partner [49]. Another study in Ethiopia found that factors associated with a higher likelihood of unmet need for modern family planning included lack of knowledge about vertical transmission of HIV and not discussing family planning with their partner [50]. ’Similarly, married women who tested positive for HIV had a high unmet need for family planning, which was associated with low education level, limited female autonomy, lack of family planning counseling in antiretroviral therapy (ART) clinics, and insufficient family planning options [51]. A common pattern observed in these studies is women’s inability to access information about family planning and lack of awareness regarding available family planning options. These findings further emphasize the need for more robust integrated services where women and their partners can seek holistic care that encompasses their sexual and reproductive health and includes family planning, STI and HIV prevention and protection against unintended pregnancies. Addressing these factors in a preventative, upstream manner would enable better management and prevention of downstream consequences, such as continued HIV transmission and high rates of unintended pregnancies in SSA.

In Fig 2, we observed that the %mCPR remained consistently higher in women who tested positive for HIV in Ethiopia and in Guinea compared to all women. Our finding regarding Ethiopian women aligned similarly with existing literature, as another study reported that married women who tested positive for HIV had a higher uptake of modern contraception compared to the general population [52]. This finding was attributed to the fact that there was an enhanced focus on care for women who tested positive for HIV in the country, with available counseling services, provision of modern contraception, and additional healthcare follow-up services [52]. In Guinea, 49% of women and 70% of men aged 15–49 were aware that condom use can reduce the risk of HIV/AIDS [53]. It is important to note that some women reported using modern contraceptives to achieve weight gain for cosmetic reasons [54]. This factor may play a role in the higher uptake of modern contraceptives among Guinean women. Additionally, the introduction of the national family planning program in Guinea in 1983, followed by its subsequent integration into public health policies and programs in 1990 [55], and continued efforts to strengthen family planning initiatives through the provision of family planning and HIV/AIDS counseling and treatment could be other contributing factors [55].

### Implications for research and policy

Our study yielded important insights into the future projections of mCPR, contraceptive prevalence rate, unmet need, and demand satisfied by modern methods for ten countries in SSA. These findings carry significant policy implications and provide valuable foresight to create effective integrated programs that target the specific needs of each country. Notably, our findings indicated that none of the ten countries included in our analyses would experience a significant reduction in the unmet need for modern contraceptive methods by 2030. This suggests that policies and programs require strengthening and tailoring to identify gaps and continued barriers that are driving unmet need. Within the literature, a pervasive health disparity exists in the lack of education and knowledge surrounding HIV awareness and appropriate family planning options. Therefore, it is essential to address this by strengthening educational resources to equip better and empower individuals to make safer sexual and reproductive health choices. Education is a powerful tool in the fight against HIV/AIDS [56,57]. Enhancing reproductive health education is an essential preventative approach to reduce unintended pregnancies and associated maternal, neonatal and child mortality rates [4,58,59].

Moving forward, countries should focus on offering comprehensive sexual education (CSE) and prioritize the development of educational resources and tools that focus on both HIV and pregnancy prevention. CSE has been identified as an important educational and preventative approach to teaching youth about sexual health [60]. It has been an important factor in the reduction of unintended teenage pregnancy in Zambia [61]. Offering CSE should be a key area of focus for healthcare centers, educators, government players, and policymakers for two reasons. First, this approach could help reduce unintended pregnancy, thereby allowing girls to stay in school longer, as studies have reported that girls are more likely to drop out of school if they have a child during their teenage years [62,63]. Remaining in school would give women more opportunities to seek employment, earn money, and gain greater independence over their finances and health decisions. Second, implementing educational programs in schools from an early age would provide youth with the opportunity to learn about safe intercourse, HIV prevention and protection, and appropriate modern family planning methods available to them. This proactive approach would help mitigate HIV transmission and unintended pregnancy rates in the next generation.

Mobilizing public health messaging through mass media is shown to be an effective method of disseminating health information [64]. It is also an important strategy to circulate educational information about sexual and reproductive health in SSA efficiently [65]. Our study projected that the unmet need for married women who tested positive for HIV (compared to unmarried women) would remain higher in 2030. In SSA, we recommend focusing on integrating educational tools and resources into the service strategy to provide sexual and reproductive health counseling and education equally to men and women. Including men in conversations around family planning and HIV awareness, testing and treatment would help to challenge the pervasive gender inequities and patriarchal structures often present in SSA, which act as barriers to health equity and prevent women from making autonomous health decisions. To strive for gender equality and promote improved reproductive healthcare access for all, the FPET is an asset. It can provide insight into future health services gaps and patterns of reproductive healthcare needs in each country. Moreover, it can help inform the creation of stronger, sustainable, integrated programs that provide a broad spectrum of sexual and reproductive health services to better address the unique needs of subgroups around the globe. By anticipating future family planning needs with the FPET, healthcare professionals, governments, and policymakers can take intersectoral action to strengthen health systems at all socioecological levels. Participatory approaches incorporating iterative feedback from relevant stakeholders and knowledge users are essential to ensure the creation of culturally relevant and sustainable integrated programs and services. This process will enable these programs to survive scale-up and receive buy-in from all stakeholders.

### Limitations

There are a few limitations to our study. First, we could not include all SSA countries in the analysis due to data limitations. Countries such as Kenya, where the HIV prevalence is high, were excluded due to lack of data points. However, our results are useful for policy and program implementation in the included countries. Further, our results can be updated and replicated in other SSA countries when more data points are available. Second, our estimation process did not account for systemic shocks such as pandemics that might affect HIV and contraceptive prevalence. Third, we did not account for any deviations in HIV prevalence due to advancements in treatment available for future projection. Future research should address these limitations to derive accurate predictions for SSA and other nations. Fourth, we only estimated the rates rather than the actual numbers for projections. This limitation arose from model assumptions that did not accommodate changing population distributions. Lastly, we did not include service statistics that could help derive program-related output variables and improve predictions. Unfortunately, we could not locate suitable service statistics to supplement our analysis. Nevertheless, our results should be updated whenever such statistics become available.

## Conclusion

Our study projected that the unmet need for modern contraception among married women who tested positive for HIV would likely remain higher in 2030. The estimated unmet need for modern contraception was particularly notable in Cameroon, Senegal, and Sierra Leone. These countries were projected to have the highest unmet need for modern contraception for those married and unmarried, respectively. It will be essential to ensure that upstream mitigation strategies are implemented proactively to address this need. We anticipated that if countries adhere to their current policies and initiatives until 2030, it will be more likely that HIV-positive married women will attain significant coverage compared to unmarried women who tested positive for HIV. These findings provided important insights into future projections for the unmet need for modern contraception among the ten countries included in this study. Our projections emphasized the importance of country-specific context when implementing initiatives and crafting policy to ensure contextual relevance and sustainability. Strengthening initiatives, programs, and services targeting unmarried women should be a primary focus to address this gap.

## Supporting information

S1 Table List of sub-Saharan countries and DHS survey data used for analysis. (DOCX)

S2 Table Prevalence and future projections of the use of modern contraceptives along with their demand satisfied, unmet need for women who tested positive for HIV (15–49 yrs.) residing in selected Sub-Saharan African Countries (disaggregated by women’s marital /in-union status). (DOCX)

S1 Text Letter from DHS approving request for retrieval and analysis of DHS surveys. (DOCX)
