# Supplementary material for: Using the Family Planning Estimation Tool (FPET) to assess national-level family planning trends and future projections for contraceptive prevalence and associated demand for HIV-infected women in sub-Saharan Africa
Source: PLOS Glob Public Health. 2024 Aug 6;4(8):e0002637. doi: 10.1371/journal.pgph.0002637 (PMC11302922; doi:10.1371/journal.pgph.0002637)
Supplement: S1 Table — (DOCX) [file pgph.0002637.s001.docx]

**Supporting information**

S1 Table: List of sub-Saharan African countries and Demographic Health Survey (DHS) data used for analysis

| **Country** | **DHS Survey Years** |
| --- | --- |
| Cameroon | 2004, 2011, 2018 |
| Ethiopia | 2005, 2011, 2016 |
| Guinea | 2005, 2012, 2018 |
| Lesotho | 2004, 2009, 2014 |
| Malawi | 2004, 2010, 2015-16 |
| Rwanda | 2005, 2010, 2014-15 |
| Senegal | 2005, 2010-11, 2017 |
| Sierra Leone | 2008, 2013, 2019 |
| Zambia | 2007, 2013-14, 2018 |
| Zimbabwe | 2005-06, 2010-11, 2015 |
| **Total Surveys** | **30** |
| **Note:** Mali couldn’t be included despite having three DHS surveys, as it was not possible to merge HIV prevalence data with women’s questionnaire data. The first DHS round (2001-02) for Zambia was excluded from the analysis for the same reason. | |
