# Supplementary material for: Using the Family Planning Estimation Tool (FPET) to assess national-level family planning trends and future projections for contraceptive prevalence and associated demand for HIV-infected women in sub-Saharan Africa
Source: PLOS Glob Public Health. 2024 Aug 6;4(8):e0002637. doi: 10.1371/journal.pgph.0002637 (PMC11302922; doi:10.1371/journal.pgph.0002637)
Supplement: S2 Table — (DOCX) [file pgph.0002637.s002.docx]

**Supporting information**

S2 Table: Prevalence and future projections of the use of modern contraceptives along with their demand satisfied, unmet need for women who tested positive for HIV (15-49 yrs.) residing in selected Sub-Saharan African Countries (disaggregated by women's marital /in-union status)

| **Country/**  **Population** | **A. Currently married women/ Women in a union** | | | | | | | | **B. Currently unmarried/Not in a union** | | | | | | | |
| --- | --- | --- | --- | --- | --- | --- | --- | --- | --- | --- | --- | --- | --- | --- | --- | --- |
|  | **Prevalence of Modern Methods (mCPR) (%), 2022** | **Demand Satisfied with a Modern Method (%), 2022** | **Unmet need for modern methods (%), 2022** | **Change in mCPR 1983-2022**  **(SE)** | **Prevalence of Modern Methods (mCPR) (%), 2030** | **Demand Satisfied with a Modern Method (%), 2030** | **Unmet need for modern methods (%), 2030** | **Change in mCPR 2022-2030**  **(SE)** | **Prevalence of Modern Methods (mCPR) (%), 2022** | **Demand Satisfied with a Modern Method (%), 2022** | **Unmet need for modern methods (%), 2022** | **Change in mCPR 1983-2022**  **(SE)** | **Prevalence of Modern Methods (mCPR) (%), 2030** | **Demand Satisfied with a Modern Method (%), 2030** | **Unmet need for modern methods (%), 2030** | **Change in mCPR 2022-2030(SE)** |
| Cameroon | 19.05 | 38.93 | 29.76 | 15.65 | 24.59 | 46.56 | 27.86 | 5.54 | 23.42 | 68.08 | 10.89 | 20.23 | 25.35 | 71.18 | 10.15 | 1.93 |
|  | (13.08-26.53) | (28.76-49.45) | (23.79-36.80) | (4.95) | (12.18-40.70) | (28.72-65.07) | (19.55-37.34) | (6.48) | (14.47-35.45) | (53.28-79.51) | (6.56-17.62) | (6.06) | (12.45-45.45) | (50.65-85.83) | (5.09-19.69) | (7.35) |
| Ethiopia | 38.44 | 58.18 | 27.55 | 26.78 | 45.13 | 66.05 | 22.99 | 6.70 | 15.21 | 66.49 | 7.76 | 11.62 | 17.7 | 68.5 | 8.08 | 2.49 |
|  | (26.37-50.97) | (44.78-71.18) | (20.02-35.53) | (7.33) | (26.54-64.18) | (44.61-82.36) | (13.27-34.70) | (7.89) | (8.32-25.82) | (34.83-88.59) | (1.92-25.02) | (5.63) | (8.09-34.20) | (37.63-89.39) | (2.11-27.21) | (6.60) |
| Guinea | 13.34 | 46.52 | 15.41 | 11.46 | 17.57 | 50.84 | 16.8 | 4.23 | 23.54 | 62.92 | 14.03 | 18.58 | 24.93 | 65.4 | 13.18 | 1.39 |
|  | (8.21-20.54) | (32.51-59.93) | (10.59-21.97) | (4.38) | (7.91-34.13) | (30.13-71.30) | (10.99-24.76) | (6.21) | (14.62-35.95) | (44.18-78.46) | (7.09-25.15) | (6.57) | (12.28-43.95) | (41.69-83.07) | (5.44-27.05) | (7.28) |
| Lesotho | 63.07 | 81.39 | 14.41 | 52.16 | 66.16 | 83.74 | 12.71 | 3.09 | 47.62 | 84.42 | 8.61 | 42.18 | 48.86 | 85.47 | 8.1 | 1.25 |
|  | (46.69-76.96) | (66.76-90.98) | (7.58-23.89) | (7.84) | (46.59-82.23) | (66.54-93.66) | (5.42-24.46) | (8.12) | (30.79-65.92) | (68.74-93.92) | (3.79-17.04) | (8.55) | (29.20-71.74) | (66.19-95.47) | (3.12-19.20) | (8.81) |
| Malawi | 54.2 | 72.32 | 20.65 | 51.45 | 59.76 | 78.17 | 16.79 | 5.56 | 31.75 | 76.32 | 9.72 | 27.93 | 34.42 | 77.98 | 9.3 | 2.67 |
|  | (39.20-68.02) | (57.56-83.74) | (12.85-29.79) | (6.32) | (40.00-78.26) | (58.47-91.14) | (7.53-28.92) | (8.19) | (18.86-47.57) | (61.60-88.26) | (5.12-16.96) | (6.27) | (16.61-56.30) | (58.34-91.29) | (3.94-19.27) | (8.27) |
| Rwanda | 59.54 | 82.64 | 12.42 | 59.02 | 63.98 | 85.41 | 10.96 | 4.44 | 19.52 | 70.91 | 7.96 | 18.29 | 21.88 | 72.44 | 8.47 | 2.36 |
|  | (44.90-72.32) | (70.67-90.78) | (7.20-19.02) | (5.65) | (43.61-80.84) | (68.66-94.54) | (4.59-20.51) | (8.04) | (10.89-32.28) | (54.94-83.71) | (4.07-15.14) | (5.26) | (10.19-41.67) | (51.21-86.86) | (3.47-18.05) | (7.27) |
| Senegal | 20.83 | 40.38 | 30.65 | 16.29 | 26.95 | 48.9 | 27.85 | 6.12 | 21.43 | 65.7 | 11.04 | 21.27 | 23.11 | 68.45 | 10.52 | 1.67 |
|  | (12.43-32.21) | (26.22-55.75) | (22.06-40.32) | (5.78) | (13.31-46.60) | (29.18-70.70) | (18.00-39.06) | (7.28) | (11.48-34.68) | (34.65-87.19) | (3.29-33.82) | (4.90) | (10.67-43.17) | (39.00-88.72) | (3.05-32.40) | (7.46) |
| Sierra Leone | 23.07 | 47.99 | 25.26 | 22.17 | 31.01 | 57.24 | 23.1 | 7.94 | 23.45 | 62.56 | 13.88 | 16.70 | 24.55 | 64.83 | 13.35 | 1.10 |
|  | (13.82-33.87) | (30.45-63.21) | (17.33-35.94) | (4.98) | (16.24-51.13) | (35.95-77.00) | (14.21-34.93) | (7.41) | (15.54-34.36) | (46.99-77.58) | (6.73-25.39) | (6.56) | (12.35-43.15) | (44.53-82.79) | (5.45-27.32) | (7.04) |
| Zambia | 48.49 | 73.84 | 17.01 | 45.82 | 54.85 | 78.37 | 15 | 6.36 | 26.72 | 68.51 | 12.04 | 23.54 | 29.26 | 71.11 | 11.51 | 2.55 |
|  | (38.36-58.70) | (63.90-82.29) | (12.27-22.63) | (5.90) | (37.16-71.42) | (61.95-89.96) | (7.90-24.18) | (7.39) | (16.72-38.97) | (55.24-80.25) | (7.45-19.59) | (6.11) | (15.16-48.05) | (50.33-86.11) | (5.32-22.81) | (7.43) |
| Zimbabwe | 68.29 | 85.33 | 11.67 | 54.46 | 70.31 | 86.96 | 10.47 | 2.02 | 36.41 | 84.42 | 6.54 | 28.86 | 38.36 | 85.05 | 6.46 | 1.95 |
|  | (55.31-79.69) | (75.26-92.50) | (6.48-18.60) | (8.03) | (51.90-84.84) | (72.22-95.25) | (4.21-20.68) | (7.57) | (22.58-53.05) | (71.41-92.90) | (3.43-12.08) | (7.16) | (19.53-60.37) | (67.59-94.77) | (2.82-14.98) | (8.44) |
