# Supplementary material for: Using the Family Planning Estimation Tool (FPET) to assess national-level family planning trends and future projections for contraceptive prevalence and associated demand for HIV-infected women in sub-Saharan Africa
Source: PLOS Glob Public Health. 2024 Aug 6;4(8):e0002637. doi: 10.1371/journal.pgph.0002637 (PMC11302922; doi:10.1371/journal.pgph.0002637)

**Supporting information**

S1 Text: Letter from DHS approving request for retrieval and analysis of DHS surveys


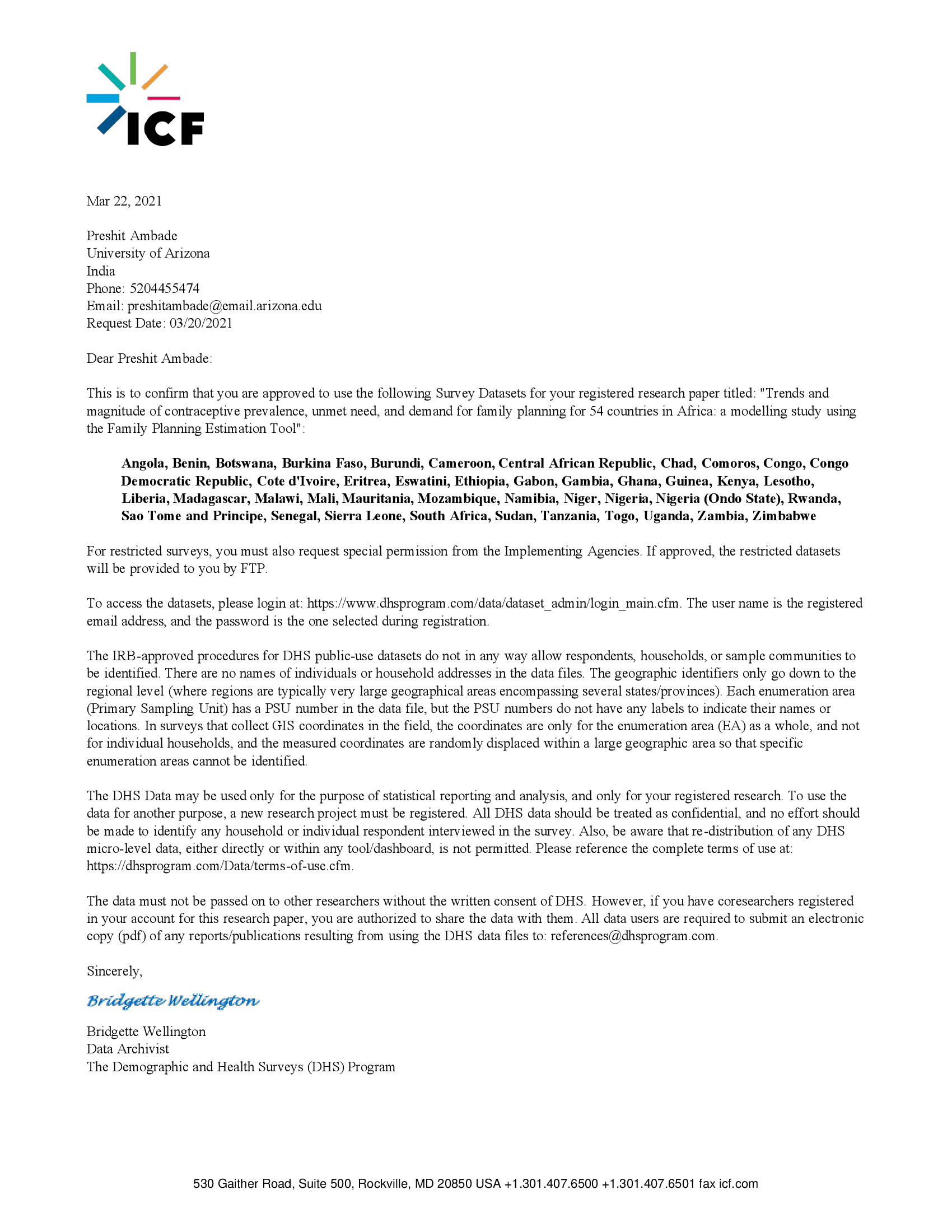

Supplement: S1 Text — (DOCX) [file pgph.0002637.s003.docx]
